# Insurance Discrimination, Companion Animal Harm, and Domestic Violence and Abuse — Double Jeopardy in the UK

**DOI:** 10.1177/10778012231176201

**Published:** 2023-05-25

**Authors:** Di Turgoose, Ruth E. McKie, Paris Connelly

**Affiliations:** 4487 De Montfort University, Leicester, UK

**Keywords:** domestic violence and abuse, insurance discrimination, companion animals, interspecies households, pet insurance

## Abstract

Prompted by Signal et al.’s study, this research examines UK “Pet Insurance” policies to see if and how experiencing domestic violence and abuse (DVA) in interspecies households is excluded under insurance policies terms. Situating our findings within the existing literature on human and companion animal victims of DVA, we discuss the implications for improving cross-reporting and multi-agency action to protect and prevent harm to humans and companion animal victims of DVA. In turn we identify a series of recommendations to combat discrimination in insurance, set out in our conclusion.

The United Kingdom (UK) is a nation of animal lovers (Campbell, 2014) who regard pets as Kin (Charles & Aull Davis, 2008) with reports indicating that 41% of UK adults “own” a companion animal (PFMA, 2021).^1^ It is now relatively well documented that companion animal abuse arises in (interspecies) households where domestic violence and abuse (DVA) takes place (Campbell et al., 2018; Faver & Strand, 2003; Riggs et al., 2021; Taylor & Fraser, 2020), whereby an abuser takes advantage of the bond between a human victim of DVA and their companion animal, often coercing them into staying out of worry for the repercussions for their companion animal should they leave, or in returning “home” to care for their animal due to lack of support for interspecies victims (e.g., Flynn, 2000). This then increases the risk of further harm being perpetrated against both the human and companion animal victims by the abuser. While there is growing knowledge of the bond between a human DVA victim and their companion animal, with some examples in practice of (a) raising awareness of the issue and (b) increase in multi-agency cooperation including support from veterinary surgeries resulting in a growing awareness to help protect and provide support to DVA victims, the provision available is still at best something of a postcode lottery.

We live in a risk society (Beck, 1992) in a world where we attempt to measure and mitigate against risk as part of everyday life. Thus, we are well versed in obtaining insurance coverage to mitigate property and health risks. It follows then that insurance against harm and injury for pets would be pursued. Indeed, almost 50% of interspecies households in the UK have pet insurance policies (Mintel, 2021). Pet insurance plans are intended to mitigate against the risk of treatment costs for injury or of a companion animal developing a health complaint which would otherwise be potentially prohibitively expensive. For us, pet insurance could also offer a protective measure to support companion animals, including those who are victims of DVA. However, as Signal et al. (2018, p. 718) note, “although the role of companion animals within the dynamic of DVA is increasingly recognized, the overlap of animal harm and insurance discrimination for victims of DVA (for interspecies households our emphasis) has not been considered.” This means that, while pet insurance may function as intended by providing a financial safeguard in the event an animal suffers an accidental injury, the insurance system may not protect victims of interspecies DVA, instead declaring claims made to be null and void, or not insuring in the first instance due to previously having been a victim of DVA, thus penalizing a victim for the harm caused by an abuser toward a companion animal in a DVA scenario.

Adopting a nonspeciesist lens, this article uses Signal et al.'s (2018) work as a starting point to examine the case of potential pet insurance discrimination in the UK in DVA scenarios. We present the results of a content analysis of “pet” and “home contents” insurance policies to determine to what extent these policies include potential exclusionary and discriminatory clauses that may penalize interspecies victims of DVA. Exclusionary measures here refer to a specific provision within an insurance policy—such as coverage for veterinary costs—that eliminates coverage at the point of claim or reduces options covered in the plan. Consequentially, the harm to companion animals and the support required by veterinary or support services regarding treatment will be voided under their terms and conditions.

Moreover, we add to the work of Signal et al. (2018) by examining more closely the potentially exclusionary measures in pet insurance as they relate to different types of DVA. Therefore, we move beyond the stereotyped physical violence gaze of DVA by examining the complexities of coercive control techniques such as financial/economic; emotional/psychological alongside physical/neglect to see how each of these elements may contribute to either (a) excluding the initial take up/purchase of policies, in other words, exclusions that may render victims of DVA “uninsurable,”^2^ (b) increase the financial costs of policies, namely charging victims of DVA a higher premium or (c) make decisions which serve to invalidate any claims made which serves to potentially re-victimize the human and companion animal DVA victim. To do this, we answer three research questions: One, do pet insurance coverage plans in the UK s exclude harms that may result from DVA? Two, do pet insurance coverage plans have any exclusionary clauses linking to different DVA forms and if so, what are they? Three, if and how do these insurance practices victim blame and what is the potential for re-victimization via these policies?

## Literature Review

### Links Between Domestic Violence and Abuse and Companion Animal Abuse

Harm to companion animals in DVA interspecies households is gaining visibility in the academic literature. While there are different elements to this area of study, the key findings of this recent attention can be broadly separated into three categories relevant here. First, consideration is given to the overlap of human and animal abuse which is often referred to as “the link” (Carlisle-Frank et al., 2004; Coorey & Coorey-Ewings, 2018; Febres et al., 2014; Flynn, 2000). That is, animal abuse (here companion animals) is viewed as a risk flag or marker for human-to-human violence, including DVA (De Gue & Dilillo, 2009). Most recently this notion of the link in DVA situations has begun to move beyond the stereotype of only comprising of physical violence. For example, Fitzgerald et al. (2020) identified the intersections between companion animal abuse and different elements of coercive control in DVA, including physical, emotional, and financial abuse. Moreover, they helped contextualize this abuse in relation to the companion animal so that such harm cannot simply be defined solely in the physical form and relegated to “property abuse.” That said, what they did not do was make links to health-related insurance.

In criminal justice related practice risk assessment and risk management reduction practice is a dominant concern in combating crime (see Kemshall, 2003 for an introduction). In DVA scenarios, criminal justice system (CJS) practitioners attempt to predict and mitigate against the risk posed to a victim by completing a DVA risk assessment screening tool “checklist,” commonly known as “DASH.” Here, one of the 24 questions asks whether the abuser has previously committed companion animal abuse (Richards, 2009).^3^ Moreover, outside of criminal justice practice and in response to the growing concern and awareness of “the link,” workshops and veterinarian training regarding animal abuse as a potential overlap with DVA has been developed (e.g., the Links Group, 23rd March 2022).

Second, scholarship has drawn attention to the pertinence of companion animals as a supporting mechanism for human victims of DVA. More specifically, research indicates that the human-companion animal bond, described as a significant relationship that can provide love and security, can play integral roles for DVA victims where closeness and safety to a companion animal can help recovery from trauma via an emotional support system (e.g., Flynn, 2000; Risley-Curtiss et al., 2010; Taylor & Fraser, 2020). So significant is this bond that human adult victims of DVA feel compelled to risk their own well-being to protect their companion animals, delay leaving, or return to, a violent relationship in an attempt to prevent (further) harm to their companion animal from the abuser (Ascione et al., 1997 Faver & Strand, 2007; Flynn, 2000; Taylor & Fraser, 2020; Tiplady et al., 2012) and more recent scholarship has begun to interpret companion animals as co-existing victims in DVA interspecies households (Riggs et al., 2021; Signal et al., 2018; Taylor & Fraser, 2020).

Existing literature and the authors own observational analysis from having worked in a DVA specialist organization on a pet DVA project, reveals that developments within practice are beginning to recognize DVA in interspecies households and are seeking to find better ways to detect, prevent and support interspecies victims. Yet, it remains that interspecies victims of DVA are often separated from each other when fleeing a DVA relationship, because of non-animal friendly shelters and landlords outdated housing tenancy contracts which prohibit the keeping of pets via a “no pets” allowed clause (despite this no longer being a requirement following a change in UK tenancy policy in 2020). In turn, this can exacerbate the trauma of DVA for both human and companion animals (Campbell et al., 2018).

Third, the risk of harm to companion animals may mean victims stay with an abusive partner and/or exacerbate the trauma if and when they leave an abusive relationship. Attention then must turn to what role financial insurance practices offer in protecting companion animals. For instance, Signal et al.'s (2018) work in Australia revealed that companion animals harmed in a household where there is DVA via nonaccidental injury often culminates in nonpay-out of an insurance policy claim. Therefore, they sought to determine whether treatment costs for injuries sustained within DVA situations (i.e., at the hands of an abuser) would be included in policies or if this would void any claim made. The results of a content analysis of Australian insurers publicly available product disclosure statements revealed exclusionary clauses in policies for women and companion animals within DVA relationships. In turn, this served as a potential form of re-victimization, with Signal et al. (2018) advocating for removing exclusionary insurance practices.

### Coercive Control in Interspecies Households

To expand on Signal et al.'s (2018) study, we situate the following discussion within the existing literature that explains the dynamics of coercive control. It is noteworthy that studies other than Signal et al. refer only to the abuse of human victims in human insurance discrimination, and not to companion animals let alone to pet insurance. More specifically, we identify a series of coercive control techniques that we hypothesize could void insurance claims; thus, resulting in discriminatory practice by insurers. Furthermore, we identify how each coercive control technique can impact on interspecies victims of DVA in a series of scenarios. Finally, while recognizing that the different techniques utilized by abusers are interrelated and are often enacted simultaneously, to aid discussion we break down coercive control techniques into the following categories: physical (and omission/neglect), behavioral, emotional/psychological, and financial/economic.

#### Physical (and Omission/Neglect) Behavioural Abuse Techniques

Signs of physical abuse to human victims of DVA can include bruising, or physical injuries including fractured bones. Likewise, veterinary practitioners can identify similar injuries in companion animals and give an informed medical assessment of whether these injuries were accidental. As Ascione (1997, p. 228) notes, abuse to a companion animal is “socially unacceptable behaviour that intentionally causes unnecessary pain, suffering, or distress to and/or the death of an animal.” This includes kicking, throwing, hitting, burning, stabbing, causing incision wounds, and perpetrating sexual abuse, administration of drugs or poison, swinging by the tail, and omission/neglect behaviors such as withholding food, medicine or access to treatment which constitute neglect (see also Lockwood & Arkow, 2016).^4^ Furthermore, we would add that limiting access to finance to obtain these resources fits in this category in so far as leading to a lack of provision of food/medication albeit unintentionally on the victim's part. This evidence of neglect then signals omissive behaviors which are potentially purposeful, that is, nonaccidental, in order to undermine the welfare of the companion animal which can also inflict further psychological and emotional harm to human companions.

Another sign of physical abuse and what could also be considered a risk indicator in DVA that needs to be considered in this context of this insurance discrimination is pregnancy and hospitalization for giving birth. Fleury-Steiner and Miller (2019) note that reproductive coercion is related to DVA’s interpersonal and psychological elements. Pregnancy can increase the isolation of a human victim, removing the autonomy to be financially independent through paid employment via work, resulting in the victim staying at home, where they can be more easily controlled. Reproductive coercion via pregnancy then, is another way that power and control in a relationship can be related to health (Chamberlain & Levenson, 2012) and we contend co-occurs with pet abuse.

#### Emotional and Psychological Abuse Techniques

Second, emotionally abusive behaviors include, making threats, verbal abuse and gas lighting (Sweet, 2019). The repeated victimization of women can also lead to further harms such as post-traumatic stress disorder (PTSD), self-inflicted harm, suicidality, and alcohol/drug abuse used as a coping mechanism for dealing with the trauma of DVA (Mazza et al., 2021). This, regarding companion animals, it is now commonly accepted and has been well documented in the literature that animals both have and can express emotions. While there are various positions in this understanding of animal emotion in its simplest form, nonhuman animals exhibit emotional experiences (De Vere & Kuczaj, 2016).

We contend that emotional abuse is aligned to the knowledge that animals are sentient beings who experience emotions. Research in animal (feminist) studies articulates how the emotions experienced by a human may be similar to and serve to impact upon that of a companion animal in a reciprocal exchange. That is, there are relational ties between the emotions of the human and their companion animal wherein both companions undertake emotional labor to support and protect each other (Taylor & Fraser, 2020). Moreover, the home is a place where animal companions may be implicated in forms of gendered DVA. Cudworth (2019, p. 426) notes “pets may be neglected or treated with cruelty or violence in this privatised space, relatively immune from public view.” Such a perspective draws on the notion of anthroparchy (Cudworth, 2011) and highlights that not only can a companion animal be interpreted as a victim of DVA, but they may also be entangled in the home, exploited, and oppressed reflecting the same social-structural conditions that supports sexism, misogyny and helps shape, foster and legitimate the perpetuation of violence against women.

#### Financial and Economic Abuse Techniques

Economic and financial abuses are also types of tactics used by an abuser to coercively control their victims. Economic abuse has been defined as “a deliberate pattern of control in which individuals interfere with their partner's ability to acquire, use, and maintain economic resources” (Postmus et al., 2018, p. 262). Financial abuse (Sharp-Jeffs, 2019) refers to the practice of an abuser to limit the ability of a DVA victim—commonly a woman—in an intimate relationship from being able to acquire, use and maintain financial resources; this should include insurance discrimination and we would contend ought to include pet insurance discrimination, but it unfortunately does not, and this is a missed opportunity to only view DVA through a speciesist lens. Littwin (2012) notes that abusive men achieve financial control over their victims by depriving access to bank accounts or by instances of coerced debt whereby credit-related transactions including loans are taken out under a ‘partner's' (victims) name either through fraudulent activities or through force.

Postmus et al. (2012, p. 411) denote that there is a “lack of understanding of economic abuse,” of its ramifications in society and with policy makers in how it interrelates, despite their research which revealed 94% of female victims experienced it. While Postmus et al. have detailed the co-occurring relationship between economic abuse and other coercively controlling techniques; for example, participants who experienced physical and psychological abuse most frequently were also subject to increased levels of monitoring, restrictions and surveillance related to the use of financial resources; what Postmus et al. have failed to do is to include insurance discrimination for companion animals in DVA into the discussion. Overlapping with the physical omissive behaviors, such as withdrawing of food and access to treatment, are financial impacts and quality of life. That is, there is a financial element where if funds are withheld the human victim may be unable to provide for their companion animals basic needs. As such, it may become the case that support for companion animals who have been a victim of DVA may be unavailable, leading to potential complications, further ailments, harm, or mortality, and other physical forms of neglect. Thus, it is paramount that economic abuse and its relationship with physical/omission behaviors (neglect) and emotional/psychological abuse is understood through an interspecies lens given that this form of coercive control impacts the human victims’ and, as their dependents, this includes companion animals.

### Insurance Discrimination

We live in a risk society where we believe that we can mitigate for hazards or *risk*s and financially *insur*e against them (see Beck, 1992). Insurance plans per se offer a form of protection against various health risks associated costs. Determining insurance plans is based on an actuarial calculation as a risk assessment to arrive at a premium based on likelihood of harm via a group (not an individual) score. Hellman (1997) explores how decisions whether to insure and at what level of cost to the individual are arrived at via these actuarial calculations, and she discusses the inherent gender bias and victim blaming mentality of such an approach applying this to human–human DVA scenarios, which is predicated on flawed lifestyle theory (see Hindelang et al., 1978 for a discussion of lifestyle theory), where the woman is seen as precipitating their own victimization by “choosing” to be a victim of DVA, and responsibilized^5^ for (largely men's) abuse because statistically women are more likely to be victims of DVA including most at risk of repeat victimization (Walby et al., 2014). While the available literature does offer some enlightenment on the issue of gender bias in health insurance (e.g., Hellman, 1997) the literature examines the experiences of human victims of DVA and health insurance but omits to consider pet related insurance in interspecies households.

Similarly, Wilson (2015) has detailed insurance discrimination for DVA victims in the USA health care system, and notes that some discriminatory practices include offensive assertions, that is, that DVA victims voluntarily choose to engage in high-risk behavior as proffered in lifestyle theory of victim precipitation (Hindelang et al., 1978) and what can be considered victim blaming, thus re-victimizing victims of DVA and perpetuating the continuation of their suffering in silence. Fromson and Durborow (1998) (also USA) also noted insurance companies denied health, life and mortgage insurance based on being a previous victim of DVA, and thus denied basic life necessities to human victims of DVA. This in turn perpetuates inaccurate myths and stereotypes about DVA victims choosing to be victims. In addition, the knowledge that a victim may not be eligible for insurance because of DVA may prevent them from leaving an abusive relationship or from reporting it.

While there have been advances made encouraging the use of protocols for medical providers to identify, treat, and refer victims of DVA, with victims likewise encouraged to use this assistance and report abuse to health practitioners, insurer practices generate concern with their reliance on basing decisions whether to insure or not on information from these help-seeking activities, that is, from the very medical records that both victims and health care providers have been encouraged to develop for the purpose of helping protect victims from further violence, as well as from court or police documents. The prospect of loss of insurance coverage will cause victims to refrain from identifying the causes of their injuries and delays help seeking.

Since Fromson and Durborow (1998) challenged the view that DVA is a lifestyle choice where one chooses to be a victim of DVA or to remain in a violent situation, little has changed in real terms. While DVA is perpetrated to achieve power and control (not for monetary purposes) insurers discriminate against victims by denying, cancelling, excluding, and rating (charging a higher premium) for health, life, and property insurance through flawed data being utilized to conduct actuarial calculations. In summary and most notably however Fromson and Durborow make no reference to interspecies households or of pet insurance. With the exception then of Signal et al. (2018) in Australia, no research has been undertaken to examine risks, insurance, DVA and companion animals worldwide.

## Methodology

### Data Sources

We first undertook an online search of insurance companies to identify all companies that offered pet insurance in the UK. To be included in this study, (a) the company had to have an online web presence, (b) the target audience had to be UK consumers, and (c) the company had to offer pet insurance cover (either as a specific, stand-alone, product or as part of a home and contents policy). The research team then collected the product disclosure statements freely available online. Data collection took place between April and June 2022. In total, there were 26 insurance policies used in our analysis. A copy of the insurers is available on request to the author.

### Coding Scheme

We created a coding scheme to assess if and how injury to companion animals was defined and covered in these policies that may have discriminatory impacts for DVA victims. To develop this coding scheme, we undertook a pilot study to determine the categories used for further coding. First, we took a random sample of five insurance documents from different insurers. Three researchers then examined the sample to identify a series of exclusionary clauses, aligned with the existing literature on DVA. Table 1 breaks down the categories that we developed and then later used to code the entire dataset. We identified six overarching codes, with each including sub-codes. We then narrowed these down and split into the following categories: economic and financial abuse, physical and omissive (neglect) behaviors, and emotional and psychological abuse to align with the existing literature on DVA.

**Table 1.** Types of Exclusionary Practice.

Exclusion	Freq (%)	Sub-codes	Example
Intentional Harm and Injury	13 (12.74%)	Harm and Injury by you Harm and injury by a family member Harm by someone looking after your pet	*“We won’t cover any claim as a result of a malicious act, deliberate injury or neglect caused by you, your family or someone looking after your pet with your permission.”* (Direct Line)
Emotional Disorders and Poor Socialisation	15 (14.70%)	Pet Emotional and Behaviour Disorders Poor socialisation and Training	“Costs *arising from vicious tendencies or behavioural problems shown by your pet…”* **(The Insurance Emporium**) *Do not cover costs of behavioural problems that could have been prevented by puppy training or socialisation” (***RSPCA Covea insurance plc)**
Hospital admissions	38 (37.25%)	General Pregnancy Related to alcohol abuse, suicide, self-inflicted injuries	*“We won’t cover—costs as a result of any hospital stay that isn’t on the advice of a doctor, specialist, or consultant—costs if the admittance to hospital is as a day case patient or an outpatient”* (**Direct Line)** “*Excluding hospitalisation of You or any member of Your family permanently residing with You as a result of pregnancy.”* (Healthy Pet) “*We will not pay any costs resulting from hospitalisation for: 1. Alcoholism, drug abuse or self-inflicted injuries…”* (**Argos Pet Insurance.)**
Registration	23 (22.54%)	Registered Address Registered Owner	*“If you or your address, or the address of your pet changes you must tell us as soon as possible as this can affect the cover we provide*” (Pet Plan) “*You must be the owner and keeper of the insured dog and cat”* **(Corinium Pet Insurance)**
Legal action payment to family or others	8 (7.87%)	Payment to another family member or carer of companion animal for property damage or physical harm Damage caused by pet left on own	*“We cannot cover legal action against you by family members or people who live with you” (**Brought by Many)*** “*Liability to other people for injury and property damage—We won’t pay—any claim that occurs because of a deliberate act or omission by you or a member of your family or household.” **(LV)***
Time between injury, onset of illness and uptake of Treatment	5 (4.9%)		“*If your pet is unwell and shows signs of an injury/illness you must arrange for a vet to examine and treat your pet as soon as possible.”* **(Co-op)** “*Death from illness or claims for any changes in your pet's health or behaviour within the first 14 days of your cover start date or any illness that develops from these changes”* (**M&S)**
Total	102 (100%)		

### Analytical Process

Following coding, we combined both a quantitative and qualitative content analysis. In quantifying the data, we calculated frequencies and percentages of insurers to identify the most common exclusionary practices and caveats that may void insurance claims made by DVA victims. By drawing on qualitative examples we highlight further insight into how these draw connections with the existing literature on coercive control. Combining both forms of analysis allowed us to understand better what this means in the context of DVA and insurance discrimination and advance our understanding of pet insurance policies and discrimination against DVA victims.

## Findings and Discussion

Our analysis revealed there were six areas of exclusion clauses that may indirectly lead to a form of insurance discrimination against victims of DVA. The following section breaks down each of these exclusions drawing on how they relate to the existing literature on DVA with Table 1 providing examples. The six categories we identified were Hospital Admissions, Physical harm and injury, Emotional disorders and poor socialization, Time between injury or the onset of illness and uptake of Treatment, Registration at a home address or DVA victim, and Legal Action or Payment to Family or others to compensate for damages caused to property. Moreover, all these exclusions can directly or indirectly affect the companion animal and human victim of DVA.

### Hospital Admissions

The most common exclusion clause centers on hospital admissions (*N* = 38, 37.25%). Under this category, there were three sub-codes: (a) emergency/unexpected admittance to hospital, with a view that companion animals would be cared for in a pre-planned hospital visit, (b) hospital admissions related to alcoholism, drug abuse, attempted suicide and/or self-inflicted injury, and (c) hospital admissions related to pregnancy and birth. Under the category of general hospital admissions, the key issue is hospital admissions under 4 days and the use of day and outpatient services. For instance, Direct Line states, “*…*We won’t cover costs if the admittance to hospital is as a day case patient or an outpatient.” Note, they will cover only more than a 4-day in-patient stay, and in reality, victims of DVA are most likely to present at Accident and Emergency Departments and be made subject to day and outpatient care.

While clinicians and hospital staff may have some awareness of identifying DVA, and clinicians and hospital staff may make routine enquiries during a hospital visit related to DVA, research points to a lack of awareness of the complex nature of how DVA is operationalized alongside having limited time to investigate indicators of “abuse” in a short visit (Rhodes & Levinson, 2003). With repeated short stay visits a pattern of ongoing DVA may become more evident with a pattern emerging, however, this does not mean it will be acted upon. Certainly, the authors anecdotal evidence is that health clinicians do not routinely enquire about pets when suspecting DVA (Turgoose & McKie, 2021b).

Several insurance policies excluded hospital admissions and support for pet boarding costs if the admission is for alcohol or drug abuse, attempted suicide and/or self-inflicted injury. For instance, Argos Pet Insurance states: “We will not pay any costs resulting from hospitalisation for: 1. alcoholism, drug abuse or self-inflicted injuries…” Similarly, Asda Bank inserts the clause *“*Will not pay Costs as a result of you being hospitalised due to alcoholism, drug abuse, attempted suicide or self-inflicted injuries.” Across the academic literature, researchers have found victims with a history of interpersonal violence and/or experiencing abuse will engage in self-harming behaviors as coping mechanisms, that is, they are indicators of trauma (e.g., Cafferky et al., 2018; Dalton et al., 2019; Hinsliff-Smith & McGarry, 2017; Sansone et al., 2007). As a result, such clauses could serve to financially burden a victim of DVA whose hospital admission is beyond their control, that is, the victim is responsibilized for the perpetrators abuse, and thus victims have no choice but to pay toward boarding costs or their companion is left at further risk if they are unable to provide care.

Lastly, some insurers do not cover the costs of hospital admissions related to pregnancy and giving birth. For example, The Equine and Livestock Insurance Company Limited state that their policy “Excludes boarding cover for pregnancy.” Similarly, Healthy Pets states, “Excluding hospitalisation of You or any member of Your family permanently residing with You as a result of pregnancy.” As aforementioned, existing literature indicates an abuser may use reproductive coercive techniques (e.g., Fleury-Steiner & Miller, 2019) to keep a woman pregnant. This coercive technique is both physical, psychological/emotional and can have economic and financial implications by removing women’s autonomy, forcing them into isolation during the pregnancy. Furthermore, when the time comes to give birth, the implications for the companion animal who may suffer at home without nutrition or support from boarding, or the financial burden placed on the human victim to cover the costs of boarding, exacerbate this sequence of oppression and harm. As a result, both the human and companion animal victim are further harmed if they are not compensated or protected in the case of boarding during a stay in hospital for pregnancy, including giving birth.

### Physical Harm and Injury

The category of physical harm by a family member was another common exclusion cause (N = 13, 12.74%). Under this category we identified the following three clauses: Harm and Injury by You, Harm and injury by a family member, and Harm by someone looking after your pet. For instance, Tesco Pet Insurance stated, “We do not pay for any injury or death to you or any member of your family or anyone else who lives with you.” Similarly, Direct Line Insurance stated, “We won’t cover any claim as a result of a malicious act, deliberate injury or neglect caused by you, your family or someone looking after your pet with your permission.” An abuser may physically harm an animal as part of the coercive controlling practices to control and manipulate their victims. Pet insurance plans which exclude, without veterinary review, the harm caused by an abuser (i.e., family member) may burden the victim financially for the claim. Moreover, the use of the term family member is significant here. This is because, it can relate to family members such as children who may have been coerced into harming their pet by the abuser (see De Gue & Dilillo, 2009).

### Emotional Disorders and Poor Socialization

Emotional disorders and poor socialization in companion animals were another form of exclusionary clause in several policies (*N* = 15, 14.70%). For example, Marks & Spencer's (M&S) insurers inserted the clause, “Policy does not cover any vet treatment for behavioral, mental or emotional disorders.” Similarly, Exotic Direct states, “We will not pay Any costs relating to mental or emotional disorders.” Emotional disorders in animals such as PTSD and generalized anxiety disorders can come from neglect, exposure to an event or ordeal, experience of violent assaults to name a few (Haq, 2017). As such, it is plausible that the harm inflicted on the animal as a victim of DVA will have long term emotional implications that are beyond something the human victim could have prevented.

On socialization, the RSPCA insurer inserts the clause, “We do not cover costs of behavioural problems that could have been prevented by puppy training or socialisation.” Not only is a human victim isolated, kept within the home and prevented from interacting with others, the same happens to companion animal victims of DVA. Removing opportunities for socialization may lead to aggressive tendencies or other signs of emotional deficits in the companion animal (Howell et al., 2015). Moreover, the notion that an owner should have supported more puppy training and increased the companion animal's socialization is a further example of responsbilizing the victim of DVA when the human victim is isolated and prevented from leaving home. We contend that this can become a form of victim blaming by placing the sole onus on the victim of DVA to control the socialization and training of their companion animal.

### Time Between Injury, Onset of Illness and Uptake of Treatment

Under this category, we identified two specific sub-categories: (a) out of hours and (b) delay in seeking treatment. Both sub-categories reflect the timeline between harm and injury and access and uptake of treatment, with the clauses causing potential significant financial barriers on the human victim and in exacerbating injuries to companion animals from delays in treatment being sought. The clause based on Out of Hours, mentioned in twelve policies, identifies that any emergency access outside of normal veterinary hours will not be covered unless later supported by a vet. For example, the insurer Exotic Direct identifies that they, “Will not pay for Extra costs for treating Your pet outside usual surgery hours, unless the Veterinary Surgeon believes an emergency consultation was necessary.” The veterinarian's judgment ultimately determines payment for out of hours care to assess the level of harm to a companion animal. If that animal requires out of hours care, they will then inform insurance companies that the call was necessary. In a study by Monsalve et al. (2017) however, they note that despite some education and awareness, veterinary practices are unclear what else they can do to address and acknowledge DVA in contributing to DVA prevention given “the link” that the harm to the companion animal may be a precursor to that of human victims (i.e., it is a red flag indicator) remains underexplored in this domain.

In the case of delayed veterinary treatment, the ability to access immediate care for an animal may be hindered by the isolation of the human victim by an abuser. Policy clauses such as those by Sainsbury's insurers state, *“*any treatment for accidental injury or poisoning which occurs or shows symptoms within 3 days of the start date,” may then financially burden the victim and potentially cause further emotional stress and companion animal suffering. Suppose that a DVA victim is unable to immediately seek medical treatment, companion animals may suffer further, injuries may be exacerbated, and the costs of treatment may increase due to delay, alongside the event that the claim could be voided. However, the isolation of a human victim of DVA is beyond their control. Thus, the victim is being responsibilized for the abusers’ behavior which again, we contend is a potential form of victim blaming.

### Registration

The category registration (*N* = 23, 22.54%), was divided into two, (a) registered at home address, and (b) registered owner. Of importance is recognizing that policies may be voided if the harm to the companion animal does not occur at the owner's registered address. For instance, M&S's insurers state, “You must be the owner and keeper of your pet and it must live with you at your home address shown on your schedule. You should tell us if you are going to give your pet to someone else and that person does not live with you at your home address.” When a victim of DVA flees an abusive home, they may not (are extremely unlikely) to have a permanent address (see Irving-Clarke & Henderson, 2021; Turgoose, 2016). As such, while they may have taken a pet with them, the address of the pet may no longer be that which is registered on the insurance contract.

As a consequence, any insurance claim made may be voided. A solution to this problem could be to provide confirmation of the address of any temporary accommodation, however this does serve to increase the potential risk posed to the victims of DVA, as it is commonly understood that risk is highest when a victim has recently left a relationship (Monckton-Smith, 2021; Turgoose, 2016) whereby informing an insurance company of a new address could jeopardize victim safety. Moreover, a claim may be voided if the animal is taken and looked after by a friend or relative, someone that is not the registered owner. This presents further harm to a victim who may have “given away” (insurers emphasis) their companion animal to reduce the risk of harm in a home environment, only for their companion to require veterinary costs that exacerbate the financial burden on the DVA victim.

### Legal Action Payment to Family

The final category we highlight are clauses based on payment in the case of legal action taken against the companion animal owner to recover costs caused for damages such as the destruction of property that may be associated with a companion animal behavioral disorder (see McMillan et al., 2015). For instance, “Liability to other people for injury and property damage…” (LV Insurance). A victim of DVA may mitigate the risk of any further abuse to their companion animal and move them to another household, whether family, a friend, or someone trustworthy that can care for the animal. However, suppose the animal becomes destructive or causes damage to property. In that case, that may partly be a by-product of behavioral and emotional disorders brought on by experiencing DVA, identified through cross-agency working could ensure that this exclusionary clause is relaxed. Without this, the financial burden on a DVA victim results in re-victimization and responsibilization for the abuser's behavior.

### Coercive Control and Pet Insurance Discrimination: A Paradigm

**Figure 1.**
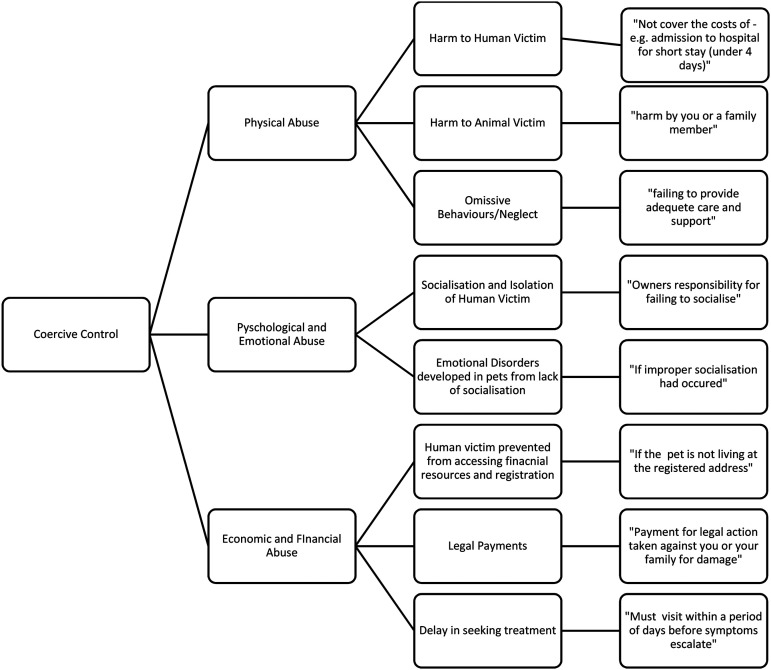
Coercive control, companion animal and insurance discrimination paradigm.

Figure 1 divides the exclusion criteria under the three categories of coercive control: economic and financial abuse, physical and omissive (neglect) behaviors, and emotional and psychological abuse. Notably these overlap, reflecting the complexity of coercive control and the need for further understanding of how companion animals are co-existing victims of DVA. On the category of economic/financial abuse we identify the following specific clauses: (a) Registration, (b) Legal Payments, and (c) Delay in seeking treatment*.* Such exclusions include costs associated with out of hours veterinary care, not paying for companion animal boarding regarding pregnancy, or self-inflicted injury (i.e., alcohol and drug abuse or self-harm) to the human, and voided claims which will not pay out to a person that may be looking after the companion animal temporarily. A solution to mitigate the voiding of policies given these circumstances is to devise fairer assessment practices not predicated on bias, victim blaming or stereotypes. This would require educational training practices including but not limited to programs on how DVA is operationalized.

In the second category of physical abuse, we combined harm to animals that included family members and significant others, including an abuser. Victims need to feel able to report nonaccidental incidents accurately, but they may not do so for fear of reprisals or due to fear of pay-out being voided and any treatment costs already honored being sought via recovery from the victim due to the victim blaming exclusion clauses used by insurers already noted earlier. This will likely result in victims in interspecies households remaining in abusive relationships.

In the final category, emotional abuse we include poor socialization and emotional disorders for an animal, which does overlap with other forms of abuse. Thus, this figure illustrates the complex interplay between different coercive control techniques and how they may manifest including unintentionally to become embedded into exclusionary clauses.

Because these clauses fail to fully comprehend and acknowledge the potential harm associated with DVA, we support Signal et al.'s, position on cross-reporting to identify the complex diverse types of harms to both human and companion animal victims of DVA. This requires adopting an intersectional awareness of coercive control that includes companion animals and how insurers and others such as veterinarian practitioners can support the broader path to end violence against women and companion animals. We believe to mitigate the negative impact of an anthropocentric approach to pet insurance discrimination and lack of awareness on the issue of DVA that may void claims or assume a human victim is a bad risk or uninsurable, underwriters should consider the following recommendations. One, underwriters must consider what information they base their decision on between claim making, and (decline of) of payments. For instance, a rethink is required in terms of what information and how they will base their decision making, that is, what medical records data to include/exclude in their calculations in future in the specific context of interspecies DVA. Two, avoid the assumptions of “uninsurable” and “bad risk” based on owner experience associated with a “Lifestyle theory” and victim precipitation that a victim of DVA places themselves and their companion animal in this situation. A point also alluded to in the work of Signal et al. (2018) and reflected in existing research on women's health insurance (e.g., Fromson & Durborow, 1998; Wilson, 2015). Three, contribute toward and engage with cross-agency reporting that could also go some way to detecting and potentially identifying offenders on DVA building up prosecutorial evidence in criminal cases and toward civil legal remedies. Four, review policies that use gender neutral language, which masks and/or potentially invisiblizes the differential impact this has on women victims of DVA and their companion animals.^6^

## Conclusion

Our study aimed to contribute to the literature on DVA and companion animal abuse whilst also looking to extend knowledge on DVA, coercive control and importantly developments on literature examining financial and economic abuse. First, and most importantly we have raised the issue and provided examples of potential discriminatory exclusion clauses in companion animal insurance in the UK answering our first research question. Second, and answering our second research question, our data indicates that exclusion criteria are largely focused on physical violence which is perhaps unsurprising given CJS practices largely centre on seeking evidence via quantifying physical harm and injury to DVA victims as opposed to nonphysical harm of DVA. This is despite coercive control legislation introduced in 2015 in the Serious Crimes Act. Nevertheless, some policy clauses were associated with nonphysical harm—that is, that is trauma arising from psychological and emotional abuse. These connect the physical, the psychological and financial harms of human victims of DVA and their companion animal; therefore, a growing awareness of the complexities of DVA coupled with the will to address it is thus needed. Finally, answering our third research question, exclusionary clauses we identified certainly contributed toward the re-victimization of DVA victims and victim blaming, and we call for a more informed assessment calculation process be undertaken by insurers which moves beyond focusing on group score static data to assess individuals, toward including dynamic clinical indicators in order to decrease the current gender bias discriminatory practices actuarial calculations are based on. For this to be realized, bias, myths and stereotypes of victimization and victim blaming of DVA victims (which are well cited in the literature) need to be addressed by a more trauma informed response by criminal justice agencies, public perceptions, and cases related to insurance (Signal et al., 2018). Here we have provided further evidence of how these exclusionary clauses potentially increase the risk of re-victimization for DVA victims and/or engage in victim blaming.

While we have outlined the problematic elements of these policies, some optimistic examples offer some way forward. For instance, LV states that they will pay for treatment/therapy of a mental or emotional disorder of a companion animal if recommended by a vet, which is an example of what could work and be effective. However, work needs to be done to alleviate/eliminate discriminatory practices and provide an educational tool that we would encourage others to use to help increase education and awareness of DVA and interspecies victims, particularly in the insurance sector.

Economic and financial abuse in our jurisdiction, has only recently come to the forefront of discussions incorporating this into debates to include in future coercive control legislation (Sharp-Jeffs, 2019) but is currently focused on human victims only. As evidenced here, this is an omission, and this research is the first to inform the gap by adding further evidence to understand the prevalence of financial abuse in coercive control legislation. Moreover, this financial abuse also impacts the companion animal victim (i.e., the inability to provide appropriate nutrition, support, and socialization for an animal). Thus, we provide further evidence to suggest the importance of recognizing and protecting animal victims of DVA which extends to the impacts of financial/economic abuse. As Postmus et al. (2012) notes, problem recognition is the first step toward finding a solution. We call for advocates and for feminist researchers to take an intersectional stance inclusive of companion animals to work together to help policy makers understand the ramifications of this problem and together form ways in which the harms caused by such discriminatory practices can be alleviated. Local and national policies designed to support victims can be expanded to acknowledge and prohibit economic abuse which includes companion animals. A first step would be to explicitly include it in guidance in the “new” Domestic Abuse Act 2022, which will recognize human–human financial/economic abuse as a distinct category of DVA for the first time.

## Notes

1. We use 2020 noting COVID-19 and the increase in companion animal ownership may not reflect existing trends as the sample size was larger given the survey was undertaken online. However, between these years there was a significant increase in pet ownership.

2. Here we refer to “bad risk,” someone viewed as “likely” to make a claim and thus not approved for insurance.

3. This is reflected in the Domestic Abuse, Stalking and Honour-Based Violence (DASH) checklist (Richards, 2009), the universal risk assessment checklist completed by criminal justice related practitioners in our jurisdiction. The DASH question is Q23: “Has … ever mistreated an animal or the family pet?” The question is asked as a red flag to indicate human abuse only so is anthropocentric.

4. Note, we use the phrase “sexual abuse” against an animal rather than bestiality because we believe the term bestiality ultimately disregards animals as sentient beings and reinforces speciesism where animals are understood through an anthropocentric lens—see Potts (2023) for a detailed examination.

5. The term responsiblizes is a well-used term in Criminology, Community and Criminal Justice where the victim is rendered responsible for an offender's actions. See Turgoose and McKie (2021a, 2021b) for an example of an explicit use of responsibilization and its reference to victim blaming in DVA.

6. We use the term invisibilize to reference the potential to “render” an act invisible. It is a term that has been recently used by feminist animal studies (FAS) academics such as Taylor et al. (2023) regarding interspecies households and companion animal harm.
